# Variety of radiological findings in a family with COVID-19: a case report

**DOI:** 10.1186/s43055-020-00293-0

**Published:** 2020-09-09

**Authors:** Farideh Gharekhanloo, Iraj Sedighi, Salman Khazaei

**Affiliations:** 1 Department of Radiology, School of Medicine, Besat Hospital, Hamadan University of Medical Sciences, Hamadan, Iran; 2 Department of Pediatrics, School of Medicine, Besat Hospital, Hamadan University of Medical Sciences, Hamadan, Iran; 3 Research Center for Health Sciences, Hamadan University of Medical Sciences, Hamadan, Iran

**Keywords:** COVID-19, Children, Chest CT

## Abstract

**Introduction:**

In December 2019, a case series of pneumonia associated with seafood and wet animal market was reported as coronavirus disease 2019 (COVID-19) in Wuhan, Hubei, China. The COVID-19 less frequently affects the pediatric group, and asymptomatic children are commonly reported. This feature is important due to the potential transmissibility of COVID-19 through these cases. There is a variety of atypical clinical symptoms and imaging manifestations among children infected with COVID-19.

**Case presentation:**

We report two sisters with COVID-19 pneumonia who had close contact with their 77-year-old grandmother. Their grandmother expired with a confirmed diagnosis of COVID-19. The older sister (15 years old) firstly presented with severe cough, fever, dyspnea, diarrhea, and lymphopenia. A chest computed tomography (CT) scan showed atypical lobar pneumonic infiltration only in the left lower lobe. The younger sister (6 years old) was affected the following day with milder symptoms (i.e., fever and cough). Her chest CT scan demonstrated typical mild generalized patchy ground-glass opacity with interlobular septal thickening scattered in both lungs. The CT presentation of their grandmother showed typical bilateral white lungs with crazy paving appearance. The standard regimen for both sisters was administrated, and the patients’ respiratory conditions improved after 5 days of treatment. Both cases were discharged with good general conditions.

**Conclusion:**

Children are considered potentially important causes of COVID-19 transmission. The symptoms in children are milder, and clinicians should be aware of taking a definite history of infected family members. Atypical clinical symptoms and imaging features are more common in children than adults; therefore, it is necessary to identify the typical and atypical imaging patterns of COVID-19 pneumonia in the pediatric population.

## Introduction

In December 2019, a case series of pneumonia associated with seafood and wet animal market was reported as coronavirus disease 2019 (COVID-19) in Wuhan, Hubei, China [1]. The COVID-19 has caused a serious outbreak in many countries. Today, the COVID-19 pandemic has posed a considerable challenge to the diagnosis and management of patients, especially in the pediatric group. There is no doubt that COVID-19 links to severe acute respiratory syndrome and Middle East respiratory syndrome less commonly affecting children. According to a published report in this regard, the prevalence of COVID-19 was less than 1% in children under 10 years of age [2]. In a cohort study carried out by Zhonghua Liu on 44,672 Chinese cases, 2.1% of the patients were under 20 years of age [3].

The neonates can be also infected by COVID-19 [4]; however, COVID-19 is less severe with milder symptoms among children in comparison to those reported for adults. Relatively few children require intensive care unit (ICU) stay, and the mortality rate among children is lower. Asymptomatic infection in children is common which is considered an important characteristic of the disease due to the potential transmissibility of COVID-19 through these cases.

There are several guidelines provided by the Radiological Society of North America (RSNA) and other institutes for typical, indeterminate, atypical, and negative CT scan patterns in adults with COVID-19 [5]. However, there have been no specific consensus guidelines on the diagnosis of suspected pediatric patients with COVID-19. In addition, the RSNA criteria are not compatible with the pediatric group and should be considered with more caution.

There have been a limited number of studies conducted on the clinical and epidemiologic features and imaging manifestations of infected children, and there have been no specific consensus guidelines on the diagnosis of suspected pediatric patients with COVID-19 [2, 6]. The current study aimed to show different patterns of children’s CT scans and compare them to adults’ CT scan patterns.

## Case presentation

### Case 1

**Fig. 1:**
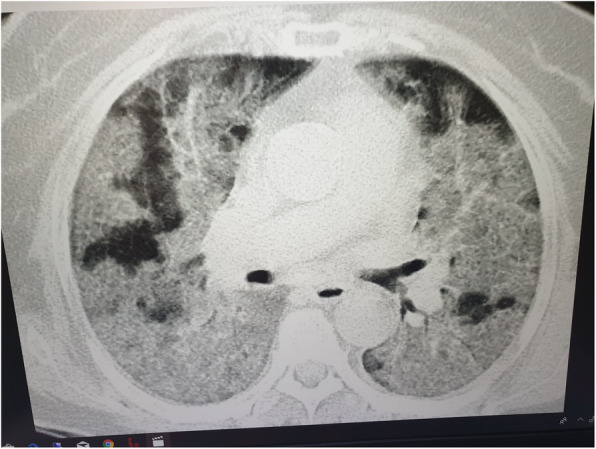
Typical bilateral peripheral ground-glass opacity (GGO) and crazy paving appearance

A 15-year-old girl was admitted to our hospital complaining of fever, dry cough, and diarrhea since 3 days ago. Her 77-year-old grandmother expired due to confirmed COVID-19 pneumonia 5 days ago. The grandmother’s disease was severe with typical bilateral peripheral ground-glass opacity (GGO) and crazy paving appearance. She died with a typical white lung in the ICU (Fig. 1). The reverse transcriptase-polymerase chain reaction (RT-PCR) of her parents was negative for COVID-19. She was reported with no underlying disorder.

At the time of admission, her vital signs showed an oral temperature of 39 °C, respiratory rate (RR) of 44 breaths/min, and pulse rate (PR) of 94 beats/min. She was dyspneic and irritable. On chest auscultation, decreased breathing sound and crepitation were heard in the left lower lung fields. She was hypoxic with peripheral capillary oxygen saturation (SpO_2_) of 82%.

Initial laboratory investigations showed a white blood cell (WBC) count of 4.9 × 10^3^/μL, lymphocyte count of 17%, neutrophil count of 75%, hemoglobin (Hb) level of 12.3 g/dL, platelet (Plt) count of 219 × 10^9^/L, erythrocyte sedimentation rate of 110 mm/h, blood urea nitrogen of 14 mg/dl, and creatinine level of 0.8 mg/dl.

The level of C-reactive protein (CRP) was elevated (CRP+++), and the level of uric acid was within the normal limit. Blood culture and polymerase chain reaction (PCR) for influenza were negative. The chest X-ray demonstrated triangular opacity in the anterolateral aspect of the left lower lung, and the computed tomography (CT) scan of the chest showed patchy segmental alveolar consolidation in the anterolateral and apical segments of the left lung lower lobe.

**Fig. 2:**
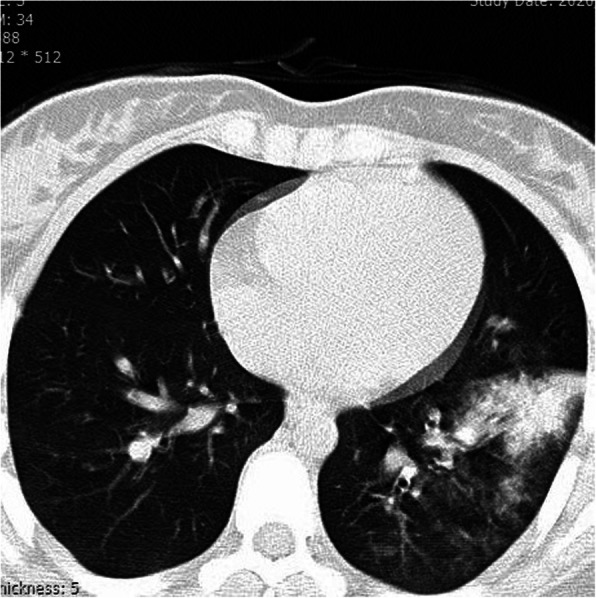
Lobes and segments of both lungs

Mild patchy density in other segments of the left lower lobe and mild peribronchial thickening in the lingula were also present. Other lobes and segments of both lungs were normal. Significant nodular density, cavitation, adenopathy, and pleural effusion were not observed (Fig. 2).

She was admitted to the pediatric intensive care unit for 2 days. According to Iranian expert’s consensus statement for COVID-19, the treatment regimen included chloroquine sulfate (daily 4 mg/kg/single dose for 5 days), oxygen, and supportive therapy [5]. After 5 days of treatment, the patient’s general condition improved, and she did not have fever, hypoxemia, tachypnea, or tachycardia. She was discharged with a good general condition.

### Case 2

One day following the admission of the first case, her sister was admitted complaining of fever and cough since 4 days ago deteriorating from the previous day. On admission, she had a temperature of 38.5°C, RR of 21 breaths/min, and PR of 88 beats/min. She was mildly dyspneic; however, severe respiratory distress was not observed. She was reported with no hypoxemia.

Initial laboratory data showed a WBC count of 7.4 × 10^3^/μL, lymphocyte count of 20%, neutrophil count of 76%, Hb level of 14.3 g/dL, Plt count of 496 × 10^9^/L, and no lymphopenia. In addition, the level of uric acid was within the normal limit. The level of CRP was elevated (CRP++). The results of blood culture and PCR for this case were similar to the findings reported for her sister.

**Fig. 3:**
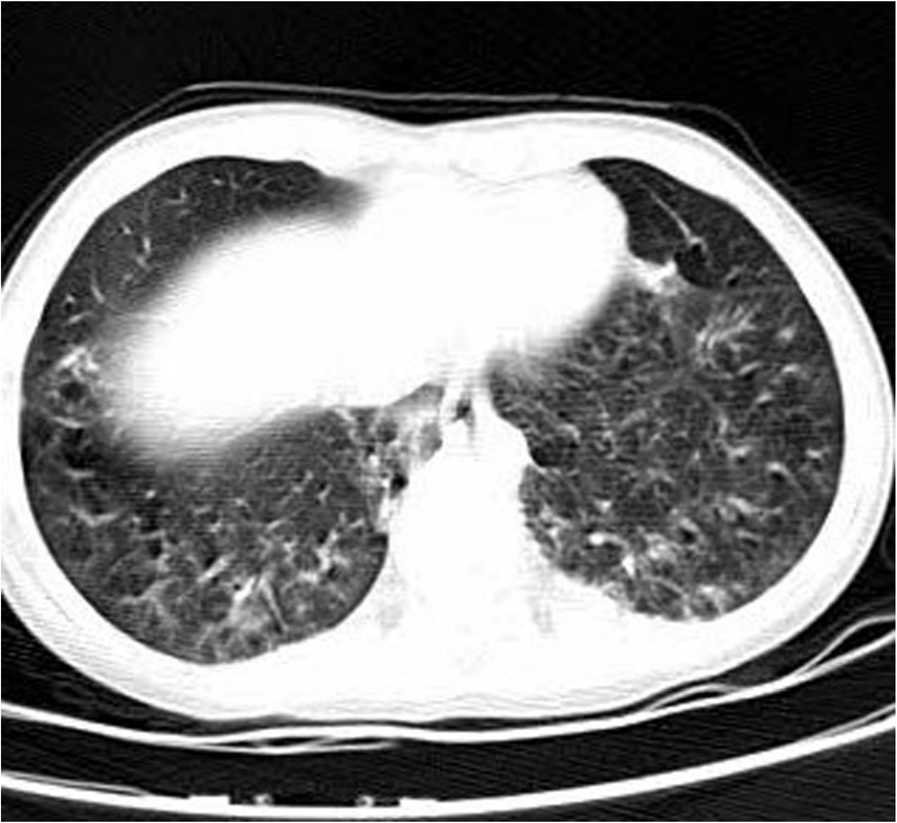
Interlobular septal thickening and reticular density in both lower lobes and right-sided pleural thickening

The chest X-ray demonstrated mild generalized GGO with interstitial reticular density. On the chest CT, symmetric patchy GGC scattered in both lungs was observed, more prominent in both lower lobes. Interlobular septal thickening and reticular density in both lower lobes and right-sided pleural thickening were also shown (Fig. 3). The treatment regimen was similar to that of her sister. After 5 days of treatment, the patient’s general condition improved. Moreover, she did not have fever, hypoxemia, tachypnea, or tachycardia. She was discharged with a good general condition.

## Discussion

According to the lower prevalence of COVID-19 among children, compared to that reported for adults, there have been no sufficient data on the clinical and imaging features of COVID-19 and its typical and atypical manifestations in children.

Based on the current criteria, laboratory data have become a standard tool for the diagnosis of COVID-19 infection. However, laboratory tests are time-consuming and a chest CT scan can be diagnostically helpful; however, the chest CT manifestations of COVID-19 pneumonia are diverse in pediatric patients [7]. Milder cases showed normal chest CT scans [8–10]. Pediatric patients showed milder symptoms and normal CT scans in comparison to those reported for adults (50% and 8.5%, respectively) [11] that may lead to missed diagnosis.

In the present cases, the older sister showed more severe symptoms. She had also rare COVID-19 symptoms, such as diarrhea. She was hypoxic with respiratory distress and required to be admitted to ICU. In addition, she had lymphopenia, and CRP was strongly positive .Her imaging pattern was predominantly compatible with focal segmental/lobar consolidation, which is considered an atypical feature of COVID-19 pneumonia according to the RSNA guidelines. The older case was treated according to Iranian expert’s consensus protocols [12].

The younger sister later became symptomatic with milder clinical symptoms. However, her imaging patterns of lung involvement were mild generalized patchy GGO and relatively mild and symmetrical distribution scattered in both lungs, with mild reticular density considered an indeterminate feature of COVID-19 according to the RSNA guidelines. However, lower lobe predominance in both cases was compatible with the lung involvement pattern of adult COVID-19. Furthermore, the absence of adenopathy, nodule cavitation, and pleural effusion was also in line with that reported for adult COVID-19.

According to the RSNA, American College of Radiology, and Society of Thoracic Radiology, there are some guidelines for CT interoperation in adults [5]. The guidelines provide radiologists with guidance for the diagnosis of adult COVID-19; however, there are no sufficient consensus criteria for pediatric patients. There are several reports on different imaging points between pediatric patients and adults.

Soltani et al. reported that the RSNA criteria cannot be generalized to pediatric patients. They also reported that only 20.7% of confirmed cases with RT-PCR showed a typical appearance of a chest CT scan. Moreover, 79.7% of these cases showed another finding considered inconsistent with CT manifestation of COVID-19. Soltani et al. emphasized that in symptomatic pediatric patients with recent close contact with COVID-19 patients, even in the presence of inconsistent appearance and atypical CT feature, the diagnosis of COVID-19 is highly probable [13].

Wei Xia et al. reported that there are different patterns of lung involvement in children. They emphasized that consolidation with a surrounding halo in pediatric patients was more common than adults, mostly due to underlying co-infection [14]. According to the overlap of COVID-19 pneumonia with other viral pathogens in a chest CT scan, they concluded that the diagnosis of COVID-19 pneumonia only by CT imaging is not enough, especially in patients with co-infection. Therefore, a chest CT scan combined with pathogen detection is a reasonable tool for diagnosis. According to a report presented by Yani Duan et al., there were more atypical CT scan manifestations in the pediatric groups, including rare interlobular septal thickening and localized GGO with a lower density of GGO [15].

Regarding the report of severe acute respiratory syndrome coronavirus 2 infection among children in the New England Journal of Medicine, out of 1391 children evaluated for the diagnosis of COVID-19, 171 cases (12.3%) were confirmed with COVID-19 infection. The most common abnormality on the chest CT scan was GGO among the cases. Local patchy shadowing, bilateral patchy shadowing, and interstitial abnormality were present in 18.7%, 12.3%, and 1.2% of the cases, respectively [16]. Therefore, this presentation is less common in children than that reported for adults.

In a study carried out by Haiyan Qiu et al. on 36 children (mean age: 8.3 years), the infection in 89% of the patients was transmitted through contact with family members; however; 33% of the subjects had a history of exposure to epidemic regions, and 47% of the cases showed mild clinical symptoms. Furthermore, less than one-third of the patients were asymptomatic, and the GGO pattern was observed in 53% of patients’ chest CT scans [17]. In another study conducted by Babyn PS et al., it was emphasized that the prevalence of pneumonia in children with COVID-19 is higher than that reported for H1N1 influenza (53% and 11%, respectively) [18].

With respect to the RSNA definition for CT scan reporting, pediatric patients more frequently demonstrate atypical and indeterminate patterns in chest CT scans. Therefore, it seems that the criteria for the application of typical and atypical in the CT scan reports of children need to be redefined.

In pediatric patients with COVID-19, a CT scan should be performed with more caution. According to the risk of radiation and high atypical manifestations of CT scans in children and risk of disease spread in hospitals by transferring the patients, CT scans should be considered for highly suspected cases with negative RT-PCR results. In addition, a CT scan should be performed according to the low-dose protocol, and CT follow-up should be reduced for such cases.

### Conclusion

The COVID-19 infection in children is less frequently common; however, mild symptoms and atypical imaging findings are possible among children. Atypical clinical symptoms and imaging features are more common among children than adults; therefore, it is required to carry out further studies with more cases for the identification of typical and atypical imaging patterns of COVID-19 pneumonia in the pediatric population. A CT scan, especially according to low-dose protocols, should be considered for highly suspected cases with negative RT-PCR results.

## Data Availability

The material of the current study is available from the corresponding author.
